# Automatic Image Recognition Meal Reporting Among Young Adults: Randomized Controlled Trial

**DOI:** 10.2196/60070

**Published:** 2025-08-14

**Authors:** Prasan Kumar Sahoo, Sherry Yueh-Hsia Chiu, Yu-Sheng Lin, Chien-Hung Chen, Denisa Irianti, Hsin-Yun Chen, Mekhla Sarkar, Ying-Chieh Liu

**Affiliations:** 1 Department of Computer Science and Information Engineering, College of Engineering, Chang Gung University, Taoyuan, Taiwan; 2 Department of Neurology, Chang Gung Memorial Hospital, Linkou Medical Center, Taoyuan, Taiwan; 3 Department of Health Care Management, College of Management, Chang Gung University, Taoyuan, Taiwan; 4 Division of Hepato-Gastroenterology, Department of Internal Medicine, Kaohsiung Chang Gung Memorial Hospital, Kaohsiung, Taiwan; 5 Healthcare Center, Department of Internal Medicine, Taoyuan Chang Gung Memorial Hospital, Taoyuan, Taiwan; 6 Digital Transformation Research Institute, Institute for Information Industry, Taipei, Taiwan; 7 Department of Industrial Design, College of Management, Chang Gung University, 259 Wenhua 1st Road, Guishan District, Taoyuan, 33302, Taiwan, 886 3-211-8800 ext 3284, 886 3-211-8500; 8 Department of Nutrition Therapy, Chang Gung Memorial Hospital, Taoyuan, Taiwan

**Keywords:** automatic food image recognition, speech recognition, artificial intelligence, usability evaluation, mHealth, image recognition, randomized controlled trial, recognition, nutrition, accuracy, efficacy, user perception, Taiwan, user interaction, vision technology

## Abstract

**Background:**

Advances in artificial intelligence technology have raised new possibilities for the effective evaluation of daily dietary intake, but more empirical study is needed for the use of such technologies under realistic meal scenarios. This study developed an automated food recognition technology, which was then integrated into its previous design to improve usability for meal reporting. The newly developed app allowed for the automatic detection and recognition of multiple dishes within a single real-time food image as input. App performance was tested using young adults in authentic dining conditions.

**Objective:**

A 2-group comparative study was conducted to assess app performance using metrics including accuracy, efficiency, and user perception. The experimental group, named the automatic image-based reporting (AIR) group, was compared against a control group using the previous version, named the voice input reporting (VIR) group. Each application is primarily designed to facilitate a distinct method of food intake reporting. AIR users capture and upload images of their selected dishes, supplemented with voice commands where appropriate. VIR users supplement the uploaded image with verbal inputs for food names and attributes.

**Methods:**

The 2 mobile apps were subjected to a head-to-head parallel randomized evaluation. A cohort of 42 young adults aged 20‐25 years (9 male and 33 female participants) was recruited from a university in Taiwan and randomly assigned to 2 groups, that is, AIR (n=22) and VIR (n=20). Both groups were assessed using the same menu of 17 dishes. Each meal was designed to represent a typical lunch or dinner setting, with 1 staple, 1 main course, and 3 side dishes. All participants used the app on the same type of smartphone, with the interfaces of both using uniform user interactions, icons, and layouts. Analysis of the gathered data focused on assessing reporting accuracy, time efficiency, and user perception.

**Results:**

For the AIR group, 86% (189/220) of dishes were correctly identified, whereas 68% (136/200) of dishes were accurately reported. The AIR group exhibited a significantly higher degree of identification accuracy compared to the VIR group (*P*<.001). The AIR group also required significantly less time to complete food reporting (*P*<.001). System usability scale scores showed both apps were perceived as having high usability and learnability (*P*=.20).

**Conclusions:**

The AIR group outperformed the VIR group concerning accuracy and time efficiency for overall dish reporting within the meal testing scenario. While further technological enhancement may be required, artificial intelligence vision technology integration into existing mobile apps holds promise. Our results provide evidence-based contributions to the integration of automatic image recognition technology into existing apps in terms of user interaction efficacy and overall ease of use. Further empirical work is required, including full-scale randomized controlled trials and assessments of user perception under various conditions.

## Introduction

### Background

In 2022, the World Health Organization [1,2] classified 2.5 billion adults (aged 18 years and older) as overweight and thus susceptible to chronic diseases associated with obesity. Failure to maintain appropriate nutrition among young people leads to a range of health issues later in life [3,4]. Young people also increasingly integrate smartphones into their daily lives, raising a growing interest in using this technology platform to deliver health-improving behavioral interventions, including healthy eating among young adults [5,6]. Such mobile health (mHealth) interventions are increasingly used to encourage healthy eating behaviors [7-10] and are increasingly popular among young users [11,12]. mHealth technologies already play a significant role in reshaping health care access [13,14] and allow for broad access to scalable and cost-effective solutions [8].

Artificial intelligence (AI)-based services are an emerging trend [15,16], with an increasingly substantial impact on various health care domains, providing enhanced accuracy, improved outcomes, and cost-effectiveness [17-19], and health care professionals have been found to hold favorable views toward AI [20,21]. Machine learning, an increasingly mature AI application, has the potential to revolutionize the mHealth domain [11,14].

### Challenges in Dietary Intake Input

An ideal mHealth app should prioritize ease of use, reliability, and long-term engagement [13,22,23]. However, manually entering dietary intake information poses significant usability challenges for mHealth app users [24], potentially resulting in inaccurate or incomplete reporting, and thus undermining the efficacy of managing healthy eating habits [16]. This raises an urgent need to minimize the operational loading of users [25,26], as the ease and effectiveness of food data entry methods have a direct and significant impact on the usability of dietary tracking applications [23]. Recent advancements in computer vision and deep learning show potential for replacing traditional input methods [27,28]. This study integrates automatic image recognition technology [29-31] into an mHealth app, which originally used voice-based inputs, seeking to improve accuracy and time efficiency in meal reporting.

### Objectives

This study assesses a parallel 2-group randomized trial designed to evaluate the relative effectiveness of a new automatic image-based reporting (AIR) app compared to the existing voice input reporting (VIR) app [32], in terms of reporting accuracy, time efficiency, and user perception. The key technological components use newly developed AI features to integrate a set of food images representing both single and mixed dishes [31].

## Methods

### General Overview of the Approach

The original app was designed to aid individuals in improving their dietary habits [33]. The previous iteration included voice inputs to enhance meal reporting, using AI services, specifically Google AI, to transcribe speech into text [32]. Users could use this feature to vocally report food ingredients, portion sizes, cooking methods, and other attributes for individual dishes. While the existing design was shown to be positive in terms of accuracy and was generally well received by users, there were concerns regarding the accuracy and time-consuming nature of completing meal reporting for an entire meal. Furthermore, in authentic dietary intake scenarios, voice reporting during meal consumption was not always convenient. Consequently, we developed the latest version to enhance the existing design.

The iterative development process was rooted in a user-centered design model [34] and included research, ideation, and implementation stages. We reviewed the relevant literature and commercially available apps, along with extensive brainstorming sessions among team members to generate diverse design ideas. AI techniques have been increasingly applied to food identification and nutrition-related applications; thus, one idea raised in the ideation stage was to allow users to take a single photo of an entire meal for analysis using AI-based recognition, rather than to process individual dishes in sequence. The initial automated meal recognition system was developed and validated using our developed AI recognition model, extracting features from convolutional neural networks [31], and tested under a laboratory setting. The results offered relatively high mean average precision for a range of dish types. The newly developed feature allowed users to upload a meal image for automatic recognition using the AI engine located on a remote server. This functionality was then integrated into the previous app version to improve convenience, accuracy, and time efficiency for meal reporting. The previous version, the voice-only reporting app, allowed users to simultaneously use verbal reporting of food names and attributes [32].

### App Implementation

The 2 apps were implemented in a 6.8-inch smartphone using the Android (Google, Inc) operating system. The AI server used in our previous research was improved to simultaneously recognize a set of multiple dishes and achieve a relatively high accuracy of food-image recognition under a laboratory setting [31]. Both apps used the Google Speech Cloud service for continuous speech recognition. The developed interfaces included user-friendly design elements, such as large-sized buttons and text, a simple layout, and high-contrast colors. Based on recommended design guidelines [32], the 2 apps shared a common interface design including the placement of buttons, text, and icons. Clear and intuitive visual cues were used to facilitate user interaction.

### App Operation

#### Overview

**Figure 1.**
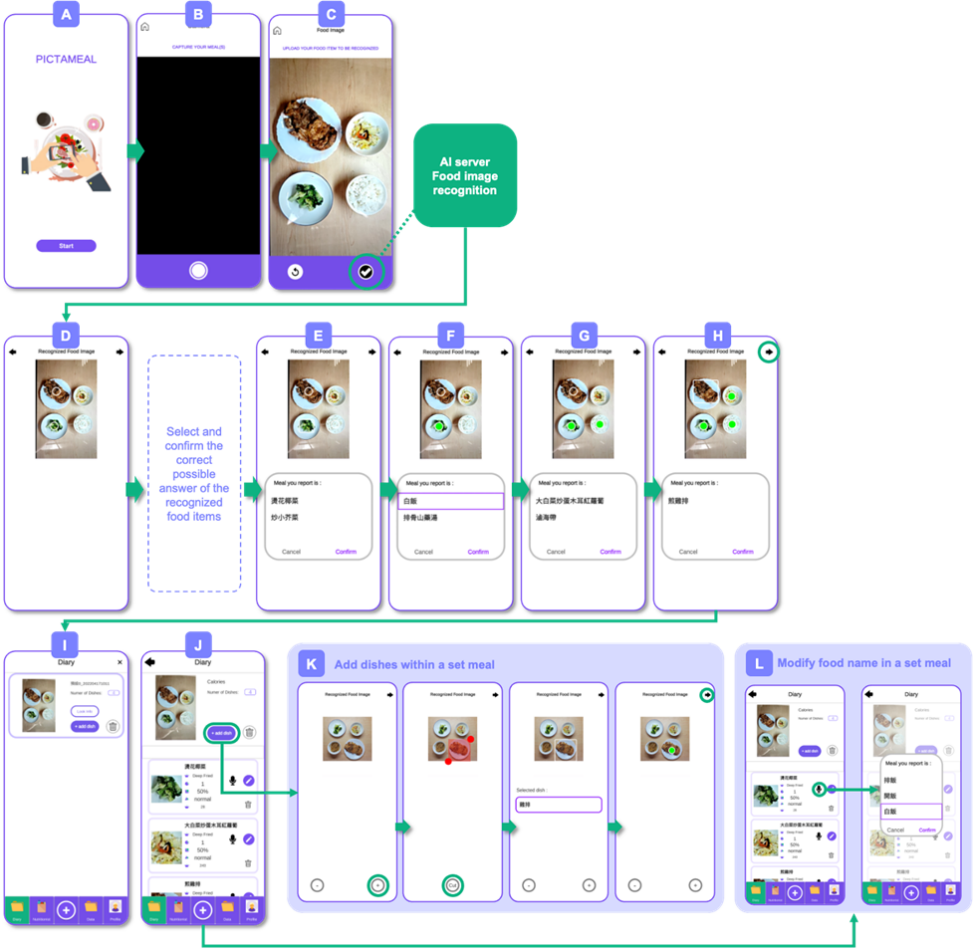
Workflow of the automatic image-based reporting (AIR) app: the user captures a food image, and the app automatically detects and works to recognize the dish (illustrated here with steamed rice as an example).

Figure 1 summarizes the 4 major stages in user interaction for the AIR design. In the first stage, users take and upload a photo of a meal (Figure 1A-C), followed by modification of the food ingredients and cooking method for each of the dishes and the provision of additional information, for example, portion size (Figure 1I,J). Users reviewed, revised, and confirmed the image, calorie content, and macronutrient information for each dish (Figure 1J,K) and were given the opportunity to add missing dishes (Figure 1J,L).

#### About AIR

To activate the app, users click the “start” button (Figure 1A) and then activate the smartphone’s built-in camera (Figure 1B). To capture the meal photo, the user clicks the icon in the lower-right corner (Figure 1C) and can retake the photo using the icon on the lower-left. Once confirmed by the user using the upload button (lower-right in Figure 1D), the meal photo is uploaded to the remote AI server for analysis. Analysis results are then sent to the app, with white dots appearing above each dish that was successfully recognized. In Figure 1, four dishes were successfully recognized (Figure 1D). Figures 1E-1H illustrate the steps by which users select the correct dish name.

For each recognized dish, up to 3 possible names with the highest confidence scores are provided (see Figure 1E). The user then selects the correct name. Upon confirmation, the color of the small circle switches from white to green, indicating that the dish entry has been completed (Figure 1E-H).

If the dish item is not recognized, or if the selection provided does not include an appropriate answer, the user resorts to an alternative input method, that is, voice input. To use the voice input method, the user drags a boundary square to surround a specific dish, then clicks the “voice” button and recites the dish name aloud (Figure 1I-K). For example, Figure 1J shows an unrecognized dish with 1 to 3 possible answers, prompting the user to respond by selecting an option using the “check” button or to return to the previous step using the “undo” button. Once all the dishes are reported, the user proceeds to the Food Diary Page to input related information such as portion size and cooking method (Figure 1J). Once dish reporting is complete, detailed information is provided for the entire meal (Figure 1L top) and for each individual item (Figure 1L bottom), including calorie count, food item name, and food attributes.

#### About VIR

The VIR process initially follows the procedure depicted in Figure 1A-C. However, rather than uploading the image to the server for automatic recognition (as depicted in Figure 1E-H), users manually manipulate the cursor (depicted as a red square in Figure 1K) to scroll down and adjust the size of the area displaying the dish image. The scrolled image is then stored for later visualization on the subsequent page (Figure 1H). Once the desired dish is identified, the user clicks the microphone icon and records the dish name. Then the user uses the voice input function to report the dish ingredients and cooking method, prompting the system to provide up to 3 options for selection and confirmation by clicking the check button, thereby concluding the reporting process. If none of the supplied answers are correct, the user clicks the microphone icon and repeats the process until the correct answer is determined. Once each dish reporting is finalized, a green circle appears above the dish. Once all dish reporting is complete, the user proceeds to the Food Diary Page to input related food information using the voice reporting process previously discussed.

### Ethical Considerations

The study protocol was reviewed by the ethics committee of Chang Gung Memorial Hospital and received institutional review board approval (202101985B0C501). A total of 42 young adults were recruited and provided informed consent. Participants received a small incentive (ie, New Taiwanese $150, roughly US $5) upon completing the study sessions. All data were handled in deidentified form to ensure participant privacy.

### Study Design and Participant Recruitment

We conducted a parallel 2-group randomized trial to compare the performance of the AIR app against the VIR app in terms of accuracy, time efficiency, and user perception. Study participant recruitment was conducted through notices placed on bulletin boards in Chang Gung University in Taoyuan City, Taiwan. Registration, schedule arrangement, and collection of background information were conducted through a web-based form. Biographic data were used to allocate participants into the AIR and VIR groups. Self-reported baseline information included gender, age, BMI, experience in nutrition education, use of nutrition-related apps, cooking experience, and experience using mobile phones or tablets. Eligible participants were (1) aged from 20 to 25 years and (2) capable of operating the app on their mobile phones. Participants currently under any form of dietary control, currently engaged in deliberate weight loss, or following a vegetarian diet were excluded. The assessment was conducted in a cafeteria at Chang Gung University.

Dishes for the experiment were selected under the supervision of a senior nutritionist (HYC) and consisted of typical local foods familiar to study participants. Foods were presented in terms of set meals involving 17 food items that were used to represent lunch and dinner. Each set meal contained 5 food items (ie, a staple food; a main course; a dish with 1 ingredient, such as stir-fried broccoli; a dish with 2 ingredients, such as stir-fried egg with tomato; and a dish with 3 ingredients).

### Sample Size Estimation

Drawing from our prior experience evaluating the young population [35], mobile app usage within this demographic exhibited nearly perfect accuracy. Consequently, in addition to assessing accuracy, we aimed to explore potential differences in time efficiency when young individuals used the newly designed app. Our previous study of customized dietary recording among young participants [35] found that the mean duration difference for the assessment of a fried pork chop was 0.7464 (2.60 vs 3.34 seconds) with SD=0.93. The sample size of that study provided a statistical power of 80% and a 2-tailed alpha level of 5%, indicating the minimum sample size required was 26 participants each for the AIR and VIR groups.

### Randomization

SAS software (SAS Institute) was used to generate randomized lists of equal size with a 1:1 ratio for the 2 studies, with 22 and 20 participants, respectively, assigned to the AIR and VIR groups.

### Evaluation Outcomes

Three outcome measures were evaluated to assess the respective performance of the 2 mobile apps. Data were collected automatically from each participant’s interaction, including the tapping of function buttons, and each interaction was logged with a timestamp. In addition, all suggested results were recorded, along with the user’s subsequent actions.

#### Accuracy

Accuracy was defined as the degree to which the response provided by the participant during app operation matched the predefined answer. Conversely, an incorrect response occurred if the app output did not match the predefined answer. The accuracy rate for a certain dish, for example, fried rice, was calculated as the overall number of correct responses divided by the total number. Furthermore, following our previous study [32], four error types were defined as follows: (1) “missing cooking method,” where the app provided an incorrect answer due to the absence of a cooking method (eg, “stir-fry”); (2) “incorrect cooking method,” where the app provided an incorrect answer due to the use of the wrong cooking method; (3) “irrelevant food name,” where the incorrect answer was due to a food name not matching the desired one; and (4) “missing food ingredients,” defined as when essential food ingredients (eg, rice) were not included in the reported answer.

#### Task Duration

The operating duration began when the participant started to report a food dish until the reporting task was completed. For the AIR group, having uploaded the image, the task duration was calculated from the time the participant clicked on the dish displayed on the screen to when the participant clicked the “complete” button. For the VIR group, the task duration was calculated from the time the participant clicked the “voice” button to begin recording to when the participant clicked the “complete” button.

#### Perception

Brooke’s system usability scale (SUS) [36] measures participant perception using a questionnaire comprising 10 items on a 5-point Likert scale ranging from 1 (strongly disagree) to 5 (strongly agree). Consistent with the approach outlined by Bangor et al [37], the mean SUS score was used alongside an adjective rating scale in which mean scores of 35.7, 50.9, 71.4, and 85.5, respectively, corresponded to the adjective scales “poor,” “ok,” “good,” and “excellent.” There is no fifth adjective category, and scores above 85.5 are generally considered “excellent.”

### Assessment Procedures

The experiment was conducted by 2 research assistants (DI and MS) who obtained informed consent from all participants. All participants used an identical 6.8-inch Android smartphone, and all trials took place in the same university cafeteria, with individual participant sessions scheduled by appointment. Before the experiment, each participant underwent training by watching an instructional video illustrating how to use the food reporting app. Each participant was allowed to interact with the app for several minutes to ensure familiarity before the trial and was informed that task completion time was included as a performance metric.

Each individual trial included 2 consecutive sessions, each requiring the reporting of a set meal. Each set meal included a staple food, a main course, a dish with 1 ingredient, a dish with 2 ingredients, and a dish with 3 ingredients. Participants were allowed to rest for up to 3 minutes between each test. The total test duration for each participant was approximately 1 hour, and all participants successfully completed the assessment.

### Statistical Analysis

Chi-square and *t* tests were applied to examine the baseline characteristics of participants for categorical and continuous variables, respectively. The accuracy between different groups was reported as the proportion of error, calculated as the number of errors or total answer items. The time duration for operating assessment was also used to evaluate efficiency. As the time duration is a continuous variable, a *t* test was used to assess and compare the difference between the 2 groups. This comparison was also applied to dishes with different ingredients. SAS (version 9.1.4) was used to conduct all statistical analyses. In all 2-tailed statistical tests, *P*<.05 was considered statistically significant.

## Results

### Participant Characteristics

**Figure 2.**
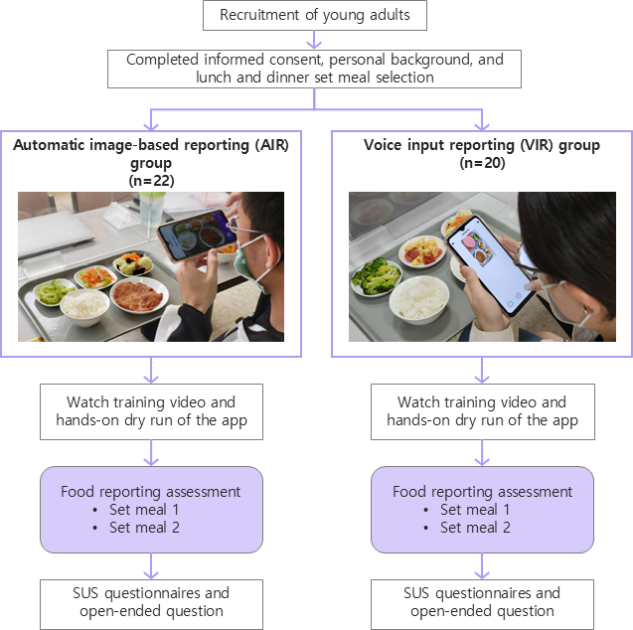
App evaluation flow using a randomized design. SUS: system usability scale.

Following the assessment procedures shown in Figure 2, all 42 participants completed the experiment, including 9 male and 33 female participants, with a mean age of 21.21 (SD 1.39) years. As shown in Table 1, 22 and 20 respondents were randomly assigned to the AIR and VIR groups.

**Table 1.** Participant characteristics of the AIR^a^ and VIR^b^ groups.

Variables	Total (N=42)	AIR (n=22)	VIR (n=20)	*P* value
Gender, n (%)				.60
Female	33 (79)	18 (82)	15 (76)	
Male	9 (21)	4 (18)	5 (24)	
Age (years)^c^, mean (SD)	21.21 (1.39)	21.32 (1.29)	21.10 (1.52)	.62
BMI (kg/m^2^)^c^, mean (SD)	21.10 (3.17)	20.50 (2.77)	21.80 (3.51)	.21
Education level, n (%)				.60
Bachelor’s degree	34 (81)	17 (77)	17 (85)	
Master’s degree	8 (19)	5 (23)	3 (15)	
Q1. Experience with nutrition-related courses, n (%)				.59
Yes	27 (64)	15 (68)	12 (60)	
No	15 (36)	7 (32)	8 (40)	
Q2. Experience with health education, n (%)				.98
Yes	23 (55)	12 (55)	11 (55)	
No	19 (45)	10 (45)	9 (45)	
Q3. Experience in cooking, n (%)				.51
Yes	39 (93)	21 (95)	18 (90)	
No	3 (7)	1 (5)	2 (10)	
Q4. Experience using nutrition-related apps, n (%)				.75
Yes	22 (52)	11 (50)	11 (55)	
No	20 (48)	11 (50)	9 (45)	

a AIR: automatic image-based reporting.

b VIR: voice input reporting.

c Age and BMI data were analyzed with ANOVA.

### Overall Accuracy

The AIR and VIR groups achieved respective overall accuracy levels of 86% (189/220) and 68% (136/200) for all 17 food dishes (Table 2). Within the food categories, the AIR group was significantly more accurate than the VIR group for the staple food (*P*<.05), main course (*P*<.05), and side dish with 3 ingredients (*P*<.05). No significant differences were found for the other food categories (ie, side dishes with 1 or 2 ingredients).

**Table 2.** Overall accuracy comparison in the AIR^a^ and VIR^b^ groups.

Type and dishes	Method		*P* value
	AIR (n=22)	VIR (n=20)	
Overall			<.001
Correct	189 (86)	136 (68)	
Incorrect	31 (14)	64 (32)	
Staple food, n (%)			
Overall			.01
Correct	43 (98)	32 (80)	
Incorrect	1 (2)	8 (20)	
Stir-fried noodle			.007
Correct	20 (95)	11 (58)	
Incorrect	1 (5)	8 (42)	
Steamed rice			N/A^c^
Correct	19 (100)	17 (100)	
Incorrect	0 (0)	0 (0)	
Fried rice			N/A
Correct	4 (100)	4 (100)	
Incorrect	0 (0)	0 (0)	
Main course, n (%)			
Overall			.04
Correct	40 (91)	29 (73)	
Incorrect	4 (9)	11 (28)	
Pan-fried chicken breast			.06
Correct	24 (92)	13 (68)	
Incorrect	2 (8)	6 (32)	
Braised pork chop			.42
Correct	16 (89)	16 (76)	
Incorrect	2 (11)	5 (24)	
Side dish with 1 ingredient, n (%)			
Overall			.06
Correct	31 (70)	20 (50)	
Incorrect	13 (30)	20 (50)	
Stir-fried eggplant			.10
Correct	14 (82)	5 (50)	
Incorrect	3 (18)	5 (50)	
Stir-fried kelp			.63
Correct	11 (85)	8 (73)	
Incorrect	2 (15)	3 (27)	
Stir-fried cauliflower			>.99
Correct	4 (50)	6 (46)	
Incorrect	4 (50)	7 (54)	
Stir-fried broccoli			>.99
Correct	0 (0)	1 (25)	
Incorrect	4 (100)	3 (75)	
Stir-fried bitter melon			.33
Correct	2 (100)	0 (0)	
Incorrect	0 (0)	2 (100)	
Side dish with 2 ingredients, n (%)			
Overall			.30
Correct	37 (84)	30 (75)	
Incorrect	7 (16)	10 (25)	
Stir-fried egg with tomato			>.99
Correct	15 (94)	14 (93)	
Incorrect	1 (6)	1 (7)	
Stir-fried loofah with carrot			>.99
Correct	9 (69)	8 (67)	
Incorrect	4 (31)	4 (33)	
Stir-fried pork with bell pepper			.03
Correct	10 (100)	5 (50)	
Incorrect	0 (0)	5 (50)	
Stir-fried bitter melon with carrot			.46
Correct	3 (60)	3 (100)	
Incorrect	2 (40)	0 (0)	
Side dish with 3 ingredients, n (%)			
Overall			.02
Correct	38 (86)	25 (63)	
Incorrect	6 (14)	15 (38)	
Stir-fried Chinese cabbage with carrot and black fungus			.11
Correct	18 (90)	11 (65)	
Incorrect	2 (10)	6 (35)	
Stir-fried cauliflower with carrot and black fungus			.46
Correct	16 (80)	11 (65)	
Incorrect	4 (20)	6 (35)	
Stir-fried bean sprout with carrot and black fungus			.20
Correct	4 (100)	3 (50)	
Incorrect	0 (0)	3 (50)	

a AIR: automatic image-based reporting.

b VIR: voice input reporting.

c N/A: not applicable.

The AIR group achieved accuracy rates exceeding 95% for individual dishes, including stir-fried noodle, steamed rice, fried rice, stir-fried bitter melon, stir-fried bitter melon with carrot, and stir-fried bean sprout with carrot and black fungus. The VIR group achieved accuracy rates exceeding 95% for stir-fried noodle, steamed rice, fried rice, and stir-fried bitter melon with carrot. The lowest accuracy for both groups was for stir-fried broccoli (less than 30% accuracy).

### Time Efficiency

Compared to the VIR group, the AIR group required significantly less time for task completion (*P*<.001), requiring around 2-25 seconds per task, with “steamed rice” being the fastest (mean 2.05, SD 1.43 seconds) and “stir-fried cauliflower with carrot and black fungus” the slowest (mean 24.90, SD 20.60 seconds; Table 3). In the VIR group, the operation time ranged from around 12 to 22 seconds per task, with “stir-fried kelp” being the fastest (mean 12.76, SD 4.12 seconds) and “stir-fried cauliflower with carrot and black fungus” the slowest (mean 21.65, SD 5.25 seconds).

**Table 3.** Dish reporting time in the AIR^a^ and VIR^b^ groups.

Dish	Reporting time (seconds)				*P* value
	AIR (n=22)		VIR (n=20)		
	n	Mean (SD)	n	Mean (SD)	
Overall	—^c^	12.43 (12.42)	—	16.25 (5.22)	<.001
Staple food	44	5.61 (6.21)	40	14.53 (5.29)	<.001
Stir-fried noodle	21	8.76 (7.42)	19	15.40 (5.75)	.003
Steamed rice	19	2.05 (1.43)	17	13.85 (5.37)	<.001
Fried rice	4	6.00 (4.90)	4	13.50 (1.76)	.03
Main course	44	9.32 (6.87)	40	15.53 (4.19)	<.001
Pan-fried chicken breast	26	6.27 (5.33)	19	16.37 (5.03)	<.001
Braised pork chop	18	13.72 (6.56)	21	14.76 (3.19)	.52
Side dish with 1 ingredient	44	12.16 (9.55)	40	15.93 (5.38)	.03
Stir-fried eggplant	17	15.06 (11.51)	10	20.40 (6.73)	.20
Stir-fried kelp	13	9.08 (6.03)	11	12.76 (4.12)	.10
Stir-fried cauliflower	8	8.00 (6.18)	13	14.54 (3.49)	<.001
Stir-fried broccoli	4	20.33 (14.64)	4	16.65 (4.25)	.57
Stir-fried bitter melon	2	14.00 (1.41)	2	18.70 (0.42)	.05
Side dish with 2 ingredients	44	15.27 (13.81)	40	15.48 (3.58)	.93
Stir-fried egg with tomato	16	12.44 (4.49)	15	15.36 (3.21)	.05
Stir-fried loofah with carrot	13	20.62 (22.68)	12	16.92 (3.63)	.58
Stir-fried pork with bell pepper	10	11.00 (4.52)	10	14.58 (3.91)	.07
Stir-fried bitter melon with carrot	5	19.00 (14.09)	3	13.33 (3.56)	.53
Side dish with 3 ingredients	44	19.80 (17.20)	40	19.76 (5.88)	.99
Stir-fried Chinese cabbage with carrot and black fungus	20	16.85 (13.35)	17	18.18 (5.52)	.71
Stir-fried cauliflower with carrot and black fungus	20	24.90 (20.60)	17	21.65 (5.25)	.53
Stir-fried bean sprout with carrot and black fungus	4	9.00 (6.22)	6	18.87 (7.91)	.07

a AIR: automatic image-based reporting.

b VIR: voice input reporting.

c Not applicable.

In the 3 food categories, that is, the staple food, main course, and dishes with 1 ingredient, the AIR group required significantly less time than the VIR group. Reporting performance for the 2 food categories did not differ significantly for dishes with 2 or 3 ingredients.

Within the 6 dishes in the categories of staple food and main course, except for “braised pork chop,” the other 5 dishes in the AIR required significantly less time than the VIR group. Within the five 1-ingredient dishes, the AIR group required significantly less time to report “stir-fried cauliflower” (*P*<.001) and was faster for “stir-fried bitter melon” (*P*<.05). For dishes with 2 ingredients, the AIR group required significantly less time in “stir-fried egg with tomato” (*P*<.05). For 3 dishes with 3 ingredients, the 2 groups did not reveal significant differences.

### SUS and Subjective Perception

Table 4 summarizes the SUS score and its 2 divisions in terms of usability and learnability. Overall scores showed no significant differences between the AIR and VIR groups, but both groups had the overall score at 84.72 and 83, respectively, indicating that the participants in both groups considered the app to be generally easy-to-use and easy-to-learn.

**Table 4.** System usability scale of the AIR^a^ and VIR^b^ groups.

Score^c^^,^^d^^,^^e^	Assessment score, mean (SD)		*P* value
	AIR (n=22)	VIR (n=20)	
Overall score	84.72 (12.66)	83.00 (11.95)	.66
Usability score	84.43 (11.34)	83.50 (9.30)	.77
Learnability score	85.00 (15.66)	82.50 (16.18)	.61

a AIR: automatic image-based reporting.

b VIR: voice input reporting.

c The questionnaire was presented in Chinese.

d Mean scores for system usability with adjective ratings are as follows: 35.7 (poor), 50.9 (ok), 71.4 (good), and 85.5 (excellent).

e The questionnaire’s Cronbach α for AIR (α=0.91) and VIR (α=0.89) exceeded 0.70, indicating good internal consistency.

## Discussion

### Principal Findings

While automatic image recognition is increasingly integrated into mobile meal reporting [28,38,39], few comparative studies have examined how this technology creates additional value for existing apps. Unlike previous studies, such as MyDietCam [26] and other existing apps [40], which primarily assessed usability through user-reported opinions or heuristic evaluation methods without using comparative experimental designs, our study used a randomized controlled trial to quantitatively evaluate differences in accuracy and time efficiency between automatic image recognition combined with voice input and voice-only reporting methods. Additional image capture techniques include wearable sensors. For example, Hussain et al [38] developed a system using a wearable camera and convolutional neural networks for food recognition.

In this study, we included VIR as the control condition because it represented the previous version of our app and served as the existing standard for comparison. At the time the study was designed, it was not known how VIR would perform relative to the newly developed AIR version. Our goal was to quantify the added value of the image recognition feature by comparing AIR against a practical and relevant baseline, namely, VIR. The results of this study indicate that combining automatic image and voice recognition is not only feasible but also provides improvements over voice-only versions in terms of accuracy and time efficiency. Our evidence-based findings provide insight for researchers or practitioners developing the next generation of dietary intake reporting applications, with implications as follows.

### Accuracy in AIR Versus VIR

Correctly identifying and reporting the dish names and cooking methods is crucial for accurate daily intake management. Table 2 shows that user performance with AIR is significantly more accurate than with VIR due to the integration of automatic image recognition. However, in the AIR group, the dishes that had been correctly recognized remain limited. Furthermore, the recognition accuracy rate of each dish for the 5 food categories fluctuated significantly within the AIR group. Image recognition errors in the trial may have resulted from the AI server failing to adequately recognize the dish or providing incorrect possible answers. In addition, errors could occur when participants were required to use voice input to complete the reporting task when the image recognition did not work properly for some dishes. Failed image recognition could also occur due to poor image quality in the uploaded file, inappropriate lighting, the technical algorithm, food recognition technologies [31,41], limited food datasets [25,26], and the contexts of use [42].

### Time Efficiency in AIR Versus VIR

Task completion for the AIR group was significantly faster than for the VIR group, suggesting that the integration of automatic image recognition can effectively reduce reporting time, though such performance improvements were inconsistent across food categories, and further enhancements are needed.

### Participant Perception

Overall, SUS results indicated participants found both apps to be easy to use. Regardless of the differences in accuracy rate and time difference achieved by the 2 groups, the app in each of the groups demonstrated high overall SUS scores.

### Limitations and Future Research

The study design assumed that users would take a photo, upload it, and report the meal in order to complete the meal reporting task. However, there are potentially other reporting scenarios to be considered in future research. For example, users may forget to take a photo before eating, or they may consume meals that are the same as or similar to previous ones. Another scenario could involve users completing the report at a later time. Adding features to the app—such as allowing users to select a previously recorded meal—could enhance flexibility and support these situations. These cases could affect the performance of automatic image recognition and warrant further investigation.

Regarding the sample size, this study was conducted during a period of severe COVID-19 restrictions, and the university was under semilockdown, making it challenging to recruit additional participants. Based on the same parameters used for the original sample size calculation, the final sample of 42 young participants yielded an estimated statistical power of 72%, which is slightly below the conventional threshold of 80%. Even in this context, the primary outcomes—accuracy and time—still showed statistically significant differences. However, these results should be interpreted with caution, as the study may have been underpowered for these measures. To generalize the findings, further confirmation with a larger and more diverse participant sample is recommended. Future research should also include a broader range of authentic dishes and longer reporting periods.

### Conclusions

Integrating AI image recognition in a voice-based meal reporting application was found to significantly improve reporting accuracy and time efficiency among young adult users. Further design improvements are required, as is testing in a broader range of authentic dining environments and a more varied array of food items.

## Supplementary material

10.2196/60070 Checklist 1 CONSORT-EHEALTH checklist (V 1.6.1).

## Abbreviations

AI: artificial intelligence
AIR: automatic image-based reporting
mHealth: mobile health
SUS: system usability scale
VIR: voice input reporting
